# Analysis of replacing DNase-seq data with histone marks in computational dimer prediction

**DOI:** 10.1186/1471-2202-16-S1-P261

**Published:** 2015-12-18

**Authors:** Victor Chukwudi Osamor, Jerzy Tiuryn

**Affiliations:** 1 Department of Computer and Information Sciences, Covenant University, P.M.B 1023, Ota, Ogun State, Nigeria; 2 Institute of Informatics, University of Warsaw, ul Banacha 2, 02-097, Warsaw, Poland

## Background

Open chromatin regions and their findings are great pointers to genome-wide revelations of transcription factor activities, which in-turn defines the level of gene participation and their roles in metabolic well-being or disease condition in human body. Chromatin openness [1] is highly influenced by the presence of histone markers which are biomarkers that define the selective modification of some amino acid at specific positions. Worthy of note is that DNase-seq useful in detecting transcription [2] activity is scarcely available in most species compared to more abundant ChIP-seq data across several species. Our interest is to investigate an optimal combination of histone marks that could replace the DNase-seq data in transcription factor dimer prediction algorithm.

## Methods

The experimental design of this work involves the analysis of the DNase-seq and all possible combinations of 5 corresponding histone markers (H3k4me1, H3k4me2, H3k4me3, H3k9ac and H3k27ac) across 3 human cell lines namely GM12878, H1hESC and K562 for a total of 31 experimental set-up. The BAM files are applied to Model-based Analysis of ChIP-Seq (MACS) [3] via its variant MACS14 to call the MACS peaks of the histone modification which is combined, sorted, merged and grouped according to base pair length not greater than thresholds starting from initial 500bp, 1000bp to 30000bp by increment of 1000bp. We are considering two kinds of regime of working with Transcription Factor (TF) dimer prediction algorithms called Transcription factor Association for Complex Overrepresentation (TACO) [4]: (1) Strongly cell-type specific (SCTS) which refers to genomic areas with chromatin opened specifically only for the considered cell-type and closed in all other cells. (2) Weakly cell-type specific (WCTS) where we consider all open area in a given cell-type without reference to other cell-types. These prepared MACS peaks data are clustered in TACO supported by 29 experimentally proven TF dimers. The narrowPeaks of DNase-seq were directly applied to TACO without any need to call their peaks or set threshold but as gold standard to assess the quality of all possible combinatorial effects [5] of histone marks under investigation.

## Results

On assessing how much quality prediction we may be compromising when we substitute DNase-seq data with histone modification in TACO, we discovered that a total of 7 experimentally proven dimers in literature were predicted correctly by TACO's WCTS algorithm in the ratio of 4:2:1 for GM12878, K562 and H1hESC cell-types respectively. This is lower than 9 experimentally proven dimers in literature obtained by our best WCTS prediction in Experiment 10 for a combination of histone modifications involving H3k4me1 and H3k9ac over 5000-7000bp. In addition, using TACO's SCTS algorithm with DNase-seq recorded 8 experimentally proven dimers in the ratio of 5:2:1 for GM12878, K562 and H1hESC respectively, while a total of 12 experimentally proven dimers were predicted in the ratio of 2:7:3 for the corresponding cell-type's trimethylation (H3k4me3) in Experiment 3.

## Conclusions

Interestingly, our result indicates that the suitable basepair range for an optimal substitution of DNase-seq with histone marks for dimer prediction may possibly be in the range of 5000-7000bp with 5000bp being the preferred. Furthermore, single modification seem to favour SCTS prediction while the WCTS tend to be more relevant for combined modifications. Our method for replacing DNase-seq with histone appears to have numerically out-performed DNase-seq and could further help in discovering chromatin openness in brain cells as well as other human cell line as applied in embryonic stem cell that could differentiate into neuro-sensory cells and organs.

## References

[B1] Cui P Li J Sun B Zhang M Lian B Li Y Xie L A Quantitative Analysis of the Impact on Chromatin Accessibility by Histone Modifications and Binding of Transcription Factors in DNase I Hypersensitive Sites BioMed Res Int 2013 2013 e914971 10.1155/2013/914971 PMC3819824 24236298

[B2] Sherwood RI Hashimoto T O'Donnell CW Lewis S Barkal AA van Hoff JP Karun V Jaakkola T Gifford DK Discovery of directional and nondirectional pioneer transcription factors by modeling DNase profile magnitude and shape Nat Biotechnol 2014 32 171 178 24441470 10.1038/nbt.2798 PMC3951735

[B3] Zhang Y Liu T Meyer CA Eeckhoute J Johnson DS Bernstein BE Nusbaum C Myers RM Brown M Li W Liu XS Model-based Analysis of ChIP-Seq (MACS) Genome Biol 2008 9 R137 18798982 10.1186/gb-2008-9-9-r137 PMC2592715

[B4] Jankowski A Prabhakar S Tiuryn J TACO: a general-purpose tool for predicting cell-type-specific transcription factor dimers BMC Genomics 2014 15 208 24640962 10.1186/1471-2164-15-208 PMC4004051

[B5] Karch KR DeNizio JE Black BE Garcia BA Identification and interrogation of combinatorial histone modifications Epigenomics Epigenetics 2013 4 264 10.3389/fgene.2013.00264 PMC3868920 24391660

